# Liver-resident CD8^+^ T cells in viral hepatitis: not always good guys

**DOI:** 10.1172/JCI165033

**Published:** 2023-01-03

**Authors:** Hendrik Luxenburger, Christoph Neumann-Haefelin

**Affiliations:** Department of Medicine II, University Medical Center Freiburg, Faculty of Medicine, University of Freiburg, Freiburg, Germany.

## Abstract

More than twenty years ago, non–HBV-specific CD8^+^ T cells were found to contribute to liver immunopathology in chronic HBV infection, while HBV-specific CD8^+^ T cells were noted to contribute to viral control. The role of HBV-specific CD8^+^ T cells in viral control and the mechanisms of their failure in persistent infection have been intensively studied during the last two decades, but the exact nature of nonspecific bystander CD8^+^ T cells that contribute to immunopathology has remained elusive. In this issue of the *JCI*, Nkongolo et al. report on their application of two methodological advances, liver sampling by fine-needle aspiration (FNA) and single-cell RNA sequencing (scRNA-Seq), to define a liver-resident CD8^+^ T cell population that was not virus specific but associated with liver damage, thus representing hepatotoxic bystander CD8^+^ T cells.

## Immunity in HBV infection

The HBV has chronically infected 260 million people worldwide, and in a substantial subset of these patients, chronic liver inflammation leads to liver fibrosis, cirrhosis, and eventually, hepatocellular carcinoma (HCC) (1). Antiviral treatment with nucleos(t)ide analogues such as tenofovir and entecavir suppresses viral replication as well as inflammation, but rarely leads to viral elimination. Indeed, functional cure of chronic HBV infection (defined as the loss of hepatitis B surface antigen [HBsAg]) may require restoration of HBV-specific immunity in addition to direct-acting antiviral strategies (2). In addition to providing a protective role in viral control, the immune response also contributes to the pathogenesis of progressive liver disease, since HBV itself is not cytopathic. It is thus of utmost importance to better understand the mechanisms of both protective immunity and immunopathology to develop optimal immune-based treatment strategies aiming at functional HBV cure without the risk of liver damage.

## Different roles of CD8^+^ T cells 

## in HBV infection

CD8^+^ T cells are important players in HBV infection. Indeed, in acute-resolving HBV infection, CD8^+^ T cells are the main effector cells responsible for viral clearance as well as disease pathogenesis (3). In chronic infection, CD8^+^ T cells contribute to partial viral control as well as disease progression. Indeed, already in 2000, Mala Maini and Antonio Bertoletti made the important observation that intrahepatic HBV-specific CD8^+^ T cells were capable of controlling HBV viremia in patients with chronic HBV infection in the absence of liver inflammation (4). HBV control was associated with a detectable, proliferative HBV-specific CD8^+^ T cell response in the peripheral blood. Patients with active HBV replication as well as liver inflammation displayed similar absolute numbers of intrahepatic HBV-specific CD8^+^ T cells; however, these HBV-specific CD8^+^ T cells were diluted due to a massive infiltrate of non–HBV-specific CD8^+^ T cells. Of note, HBV-specific CD8^+^ T cells could not be detected in the peripheral blood of these patients with active viral replication and liver disease (4). This elegant early study thus indicated that HBV control is linked to an intrahepatic HBV-specific CD8^+^ T cell response also partially recirculating to the peripheral blood, while liver disease is mostly associated with the activation of nonspecific bystander CD8^+^ T cells in the liver. In the last two decades, HBV-specific CD8^+^ T cells and the mechanisms of their failure in chronic HBV infection have been characterized in detail (5, 6). Indeed, the main mechanism that contributes to the inability of HBV-specific CD8^+^ T cells to achieve viral clearance is thought to be CD8^+^ T cell exhaustion due to high antigen loads. This concept is supported by several findings. For example, the stages of HBV infection show progressive CD8^+^ T cell exhaustion and deletion with increasing levels of exhaustion and/or deletion corresponding with increasing viral load and HBsAg levels. Similarly, nucleos(t)ide analogue therapy, which suppresses HBV replication, can partially restore HBV-specific CD8^+^ T cell function (7). Notably, different levels of CD8^+^ T cell exhaustion and deletion depend on the abundance of the targeted viral antigen (e.g., there are increasing levels of exhaustion and/or deletion in core-specific, polymerase-specific, and HBsAg-specific CD8^+^ T cells) (8–11). Another elegant study from Mala Maini’s group linked viral control in chronic HBV infection to liver-resident CD8^+^ T cells that adapted to the liver environment by expressing high levels of programmed death 1 (PD-1) and IL-2 (12). Through an autocrine feedback loop, IL-2 secretion by these cells may contribute to effective memory maintenance despite little CD4^+^ help in the intrahepatic compartment. This protective liver-resident CD8^+^ T cell population contained a high proportion of HBV-specific CD8^+^ T cells targeting all major HBV proteins (12).

## Bystander CD8^+^ T cells 

## in HBV infection

**Figure 1:**
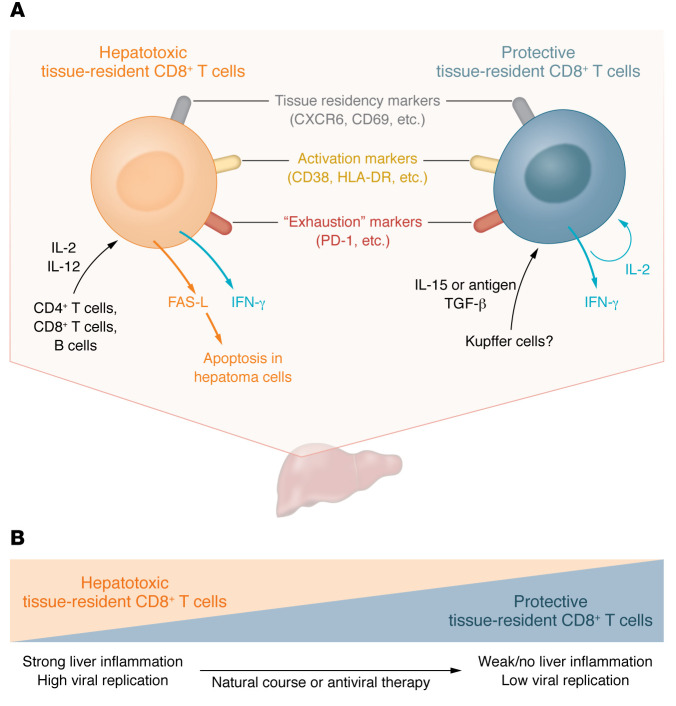
Cytotoxic and protective liver-resident memory CD8^+^ T cells define the intrahepatic immune landscape in chronic HBV infection. (**A**) Hepatotoxic and protective liver-resident CD8^+^ T cells possess common properties, such as shared surface markers, as well as divergent properties, such as differing mechanisms of induction and effector functions. In protective liver-resident CD8^+^ T cells, secreted IL-2 participates in an autocrine feedback loop. Of note, hepatotoxic liver-resident CD8^+^ T cells are not HBV specific (bystander T cells), while protective liver-resident CD8^+^ T cells include a large proportion of HBV-specific CD8^+^ T cells. (**B**) Both T cell subsets are associated with viral load and inflammation in chronic infection. Notably, antiviral therapy can shift the dominance of the hepatotoxic population to the protective population.

In contrast to protective HBV-specific CD8^+^ T cells, the exact nature and pathogenic mechanisms of nonspecific bystander CD8^+^ T cells have obtained little attention during the last twenty years. In this issue of the *JCI*, Nkongolo et al. (13) now focus on bystander CD8^+^ T cells in chronic HBV infection by taking advantage of two methodological advances: liver cell sampling by fine-needle aspiration (FNA) and in-depth cell characterization by single-cell RNA sequencing (scRNA-Seq) (13). FNA has minimal risks and causes little discomfort, allowing repeated application, and is able to provide a comprehensive picture of the intrahepatic immune landscape (14). Nkongolo et al. (13) performed scRNA-Seq in longitudinal FNAs from patients with chronic HBV infection, high viral load, and liver inflammation who were starting antiviral therapy with tenofovir. By comparing the frequencies of cell populations before antiviral treatment when liver inflammation was pronounced and at 24 weeks on treatment when liver inflammation had resolved, they identified a distinct CD8^+^ T cell population that was associated with inflammation and fibrosis. This CD8^+^ T cell population had a liver-resident memory phenotype, indicated by the expression of the tissue-residency markers CD69 and CXCR6, was polyclonal and not HBV specific, and displayed a highly activated immune signature with high expression of the effector molecules IFN-γ and FAS-L. The cells also expressed high levels of activation markers, such as CD38 and HLA-DR, and high expression of exhaustion markers, such as PD-1 and LAG-3. Through the expression of IFN-γ and FAS-L, this cell population attracted inflammatory infiltrates to the liver and contributed to apoptosis of hepatocytes expressing FAS, findings that explain its association with liver inflammation and fibrosis. The authors thus termed this liver-resident bystander CD8^+^ T cell population “hepatotoxic.” Notably, this hepatotoxic phenotype could be induced by stimulation with cytokines IL-2 and IL-12. Interestingly, IL-2 was mainly produced by intrahepatic CD4^+^ T cells and a liver-resident CD8^+^ T cell population, while IL-12 was unexpectedly produced by B cells (Figure 1).

## Bystander CD8^+^ T cells in other chronic liver diseases

It is important to note that nonspecific bystander CD8^+^ T cells are not necessarily harmful in all settings. In acute viral infection, activation of bystander CD8^+^ T cells may increase the effect of virus-specific CD8^+^ T cells and thus allow viral eradication (15). On the other hand, overwhelming bystander activation may contribute to a fulminant course of acute infection, as has been demonstrated in hepatitis A virus infection (16). In chronic viral as well as nonviral liver diseases, similar liver-resident CD8^+^ T cell populations with bystander activation have been described recently in chronic HBV and hepatitis D virus (HDV) coinfection as well as nonalcoholic steatohepatitis (NASH) (17, 18). In chronic HBV/HDV coinfection, liver-resident (CD69^+^CXCR6^+^) CD8^+^ T cells also displayed high activation (e.g., CD38) and exhaustion (e.g., PD-1) markers; however, they diverged from the CD8^+^ T cells identified as cytotoxic by Nkongolo et al. in their mode of activation, which was mediated by IL-15 rather than IL-2 and IL-12, albeit IL-15 and IL-2 are functionally related cytokines. The cell populations also displayed differences in their effector function, since cytotoxicity was mediated by NKG2D-dependent degranulation rather than FAS-L–induced apoptosis. This cell population expanded from HDV-specific CD8^+^ T cells to nonspecific bystander CD8^+^ T cells and was associated with liver inflammation (18). In NASH, a population of liver-resident (CXCR6^+^) CD8^+^ T cells was recently identified that also shared high PD-1 expression. This cell population was induced by IL-15 and was susceptible to metabolic stimuli, resulting in killing of hepatocytes in an MHC-independent manner. It was thus named “auto-aggressive” by the authors (17). Despite differences in the mode of induction and effector functions, it becomes thus more and more evident that nonspecific bystander CD8^+^ T cells with a liver-resident memory phenotype have an important role in immunopathogenesis of chronic viral and nonviral liver diseases.

## Liver-resident CD8^+^ T cells 

## as good and bad guys

It is important to note that the two populations of liver-resident CD8^+^ T cells in chronic HBV infection described by Pallett et al. (12) and Nkongolo et al. (13) may look similar at first sight, since they both express tissue-residency markers, such as CD69 and CXCR6, activation markers, such as CD38 and HLA-DR, and exhaustion markers, such as PD-1. Of note, PD-1 expression may indicate activation and/or serve as an optimal adaption to the liver’s immune landscape, allowing cell survival despite having only limited help from other cell types, such as CD4^+^ T cells. However, these two cell populations diverge strongly regarding specificity, mode of induction, effector functions, and most importantly, disease association (Figure 1A). The liver-resident CD8^+^ T cells identified by Pallett et al. include a large proportion of HBV-specific CD8^+^ T cells, are induced by IL-15 or antigen stimulation followed by TGF-β, and perform noncytolytic antiviral effector functions via IL-2 and IFN secretion (12). They are thus associated with viral control in chronic HBV infection and also persist after viral clearance (Figure 1B) (12). The hepatotoxic liver-resident CD8^+^ T cells identified by Nkongolo et al. (13), however, are non–HBV-specific bystander CD8^+^ T cells, are induced by IL-2 and IL-12, and not only express IFN-γ, but also FAS-L, which contributes to hepatocyte apoptosis and thus liver damage. Importantly, these hepatotoxic liver-resident CD8^+^ T cells are primarily found in patients with HBV replication and liver inflammation, while their quantity and activation signature substantially decreased with reduced viral replication and liver inflammation during antiviral therapy (Figure 1B). In consequence, flow cytometry analysis can identify the hepatotoxic CD8^+^ T cell population only in patients with active disease before therapy, not in patients undergoing antiviral treatment (13).

## Implications for functional cure of HBV infection

The growing understanding of the intrahepatic immune landscape, including phenotypically similar, but functionally quite divergent populations of liver-resident CD8^+^ T cells may allow targeted interventions to strengthen antiviral capacity without the side effect of increasing liver pathology. The finding that antiviral therapy largely abolishes bystander activity underscores the importance of combining direct antiviral and immune strategies aiming at HBV functional cure.

## Version 1 01/03/2023

Electronic publication
